# The influence of body composition on the N-terminal pro-B-type natriuretic peptide level and its prognostic performance in patients with acute coronary syndrome: a cohort study

**DOI:** 10.1186/s12933-016-0370-0

**Published:** 2016-04-06

**Authors:** Fang-Yang Huang, Hua Wang, Bao-Tao Huang, Wei Liu, Yong Peng, Chen Zhang, Tian-Li Xia, Peng-Ju Wang, Zhi-Liang Zuo, Yue Heng, Rui-Shuang Liu, Xiao-Bo Pu, Yi-Yue Gui, Shi-Jian Chen, Ye Zhu, Mao Chen

**Affiliations:** Department of Cardiology, West China Hospital, Sichuan University, 37 Guoxue Street, Chengdu, 610041 Sichuan China; Department of Family Medicine, West China Hospital, Sichuan University, Chengdu, China

**Keywords:** Prognostic performance, N-terminal pro-B-type natriuretic peptide, Acute coronary syndrome, Body composition, Diabetes mellitus

## Abstract

**Background:**

Whether body composition is associated with the N-terminal pro-B-type natriuretic peptide (NT-proBNP) level and its prognostic performance in acute coronary syndrome (ACS) remains unknown. We aimed to investigate the influence of body composition on the NT-proBNP level and its prognostic performance among ACS patients.

**Methods:**

In total, 1623 ACS patients with NT-proBNP data were enrolled. Percent body fat and lean mass index were estimated using the Clínica Universidad de Navarra—Body Adiposity Estimator equation. Patients were divided into three groups according to the tertiles of sex-specific body mass index, percent body fat, or lean mass index. The endpoints were death from any cause and cardiovascular death.

**Results:**

Body mass index was inversely correlated with NT-proBNP levels (β = −0.036, P = 0.003). Lean mass index, but not percent body fat, was inversely associated with NT-proBNP levels (β of lean mass index = −0.692, P = 0.002). During a median follow-up of 23 months, 161 all-cause deaths occurred, and of these, 93 (57.8 %) were attributed to cardiovascular causes. Multivariate Cox analysis showed that the NT-proBNP level independently predicted all-cause mortality or cardiovascular death in the lower body mass index, lean mass index, and percent body fat groups. However, the prognostic performance of NT-proBNP was attenuated in patients with high body mass index, lean mass index, and percent body fat. In the subgroup of patients with diabetes, inverse associations between NT-proBNP levels and body mass index or body composition were not observed. In addition, the negative influence of high body mass index and body composition on the prognostic performance of the NT-proBNP level appeared to be attenuated.

**Conclusions:**

Body mass index and lean mass index, but not percent body fat, are inversely associated with NT-proBNP levels. The prognostic performance of this biomarker may be compromised in patients with high body mass index, percent body fat, or lean mass index. Additionally, the influence of body composition on the NT-proBNP level and its prognostic performance might be attenuated in diabetic patients with ACS.

## Background

During the past decade, obesity has become a large public health problem. A previous study reported that the combined prevalence of obesity and overweight has increased by nearly 50 % from 1992 to 2008 in China [1]. A growing number of patients with obesity are at high risk for acute coronary syndrome (ACS) owing to common risk factors, such as diabetes, dyslipidemia, hypertension, and systemic inflammation. N-terminal pro-B type natriuretic peptide (NT-proBNP) is a widely used biomarker, and it has shown robust prognostic potential in ACS [2, 3]. An unexpected inverse relationship between BNP/NT-proBNP levels and body mass index (BMI) has been documented in both healthy individuals and in patients with heart failure [4], and low NT-proBNP cut-off levels are recommended in the diagnosis of heart failure among obese patients [5]. However, only two studies have investigated the relationship between the prognostic relevance of NT-proBNP and BMI in ACS patients [6, 7], and the findings of these two studies were contradictory. Lorgis et al. [6] suggested that the prognostic performance of NT-proBNP was attenuated in patients with high BMI, while Choi et al. [7] found that the prognostic performance of NT-proBNP was high in patients with high BMI.

BMI does not consider body composition and fat distribution; therefore, the use of BMI as a measure of obesity has been questioned recently [8, 9]. Body composition considers fat mass and lean mass that includes the muscles, skeleton, and body fluids. Percent body fat (%BF) and the lean mass index (LMI) have been shown to be more accurate indicators to measure obesity [8, 10]. Additionally, Das et al. [11] showed that natriuretic peptide (NP) levels were better associated with lean mass than with BMI in a healthy population. Nevertheless, the association of body composition with the NT-proBNP level and its prognostic performance in patients with ACS remains unknown. In the present study, we aimed to investigate the influence of body composition on the NT-proBNP level and its prognostic performance in patients with ACS. As obesity is generally accompanied with diabetes, we further investigated the influence of body composition on the NT-proBNP level and its prognostic performance in diabetic patients with ACS.

## Methods

### Patients

The present study used data from the West China Hospital coronary artery disease (CAD) database. This database has been described in detail previously [12]. In brief, the database included the data of consecutive patients suspected to have coronary artery disease and those who underwent coronary angiography between July 2008 and September 2012. The database prospectively collected demographic, clinical, laboratory, and treatment data of patients. The inclusion criteria of the present study were as follows: angiographic evidence of 50 % stenosis in ≥1 coronary artery with symptoms, electrocardiography or cardiac biomarker criteria consistent with ACS, presence of complete BMI data, and presence of complete follow-up information. The study protocol conformed to the ethical guidelines of the 1975 Declaration of Helsinki and was approved by the institutional review board of West China Hospital, Sichuan University (approval number 2012-243). All participants provided informed consent.

### Body composition assessment

During hospitalization, body height and weight of the patients were measured by nurses using standard methods. BMI was calculated as weight (kg) divided by the square of height (m^2^). Body fat (BF) was estimated using the Clínica Universidad de Navarra—Body Adiposity Estimator (CUN-BAE) equation: BF = −44.988 + (0.503 × age) + (10.689 × sex) + (3.172 × BMI) − (0.026 × BMI^2^) + (0.181 × BMI × sex) − (0.02 × BMI × age) − (0.005 × BMI^2^ × sex) + (0.00021 × BMI^2^ × age), where sex is replaced by 0 for male and 1 for female individuals [13]. This formula has been validated in a large population [14]. The lean mass index was calculated as follows: (1 − %BF) × BMI kg/m^2^ [9]. As no reference value has been reported in a Chinese population, we divided the study patients into three groups according to the tertiles of sex-specific BMI, LMI, or BF.

### Baseline data collection

The baseline anthropometric and blood pressure data were collected by nurses. The medical history (history of hypertension, diabetes mellitus, dyslipidemia, heart failure, cerebrovascular accident, and myocardial infarction), smoking history, vital signs at admission, echocardiographic data (left ventricular ejection fraction and left ventricular end-diastolic volume), and final diagnosis were obtained from hospital records and via interviews. In the present study, a patient was considered to have diabetes mellitus if the fasting plasma glucose level, 2-h plasma glucose level after a 75-g oral glucose tolerance test, or hemoglobin A1c level satisfied the ADA criteria at the time of presentation, diabetes was diagnosed by a previous physician, and/or the patient was using insulin or oral hypoglycemic agents. The Killip class and risk stratification of ACS based on the thrombolysis in myocardial infarction (TIMI) risk score for NSTE-ACS or STEMI were evaluated by physicians according to the presentation characteristics.

### Laboratory measurements

Venous blood samples were collected in tubes containing EDTA and analyzed at the Department of Laboratory Medicine, West China Hospital, accredited by the College of American Pathologists. Plasma NT-proBNP and TnT levels were measured using an electrochemiluminescence immunoassay kit (Roche Diagnostics, Grenzach Wyhlen, Germany). The NT-proBNP results were obtained within the first few days after symptom onset in all of the study patients. The NT-proBNP reference levels in a healthy population depend on age and sex. In male individuals, the 95th percentile level increases from 83.9 pg/mL at 45–54 years of age to 486 pg/mL at ≥75 years of age. In female individuals, the level increases from 169 to 738 pg/mL. The peak plasma TnT level during hospitalization was used in the analysis. The chronic kidney disease epidemiology (CKD-EPI) equation was used to determine the estimated glomerular filtration rate (eGFR).

### Endpoint definition and follow-up

The endpoints were death from any cause and cardiovascular death. Follow-up information was obtained by contacting patients and/or their relatives via letters or the telephone, or through outpatient visits. All data were corroborated with hospital records.

### Statistical analysis

Baseline data are presented as median (with 25th and 75th percentiles) or counts and proportions. The Chi square test or Fisher’s exact test was used to compare categorical variables across different age groups. The Kolmogorov–Smirnov test was used to assess the distribution of the data. If the data followed normal distribution, the data were compared using one-way analysis of variance; otherwise, the data were compared using the Kruskal–Wallis test. Because the NT-proBNP data followed non-normal distribution, the data were log-transformed for multivariate analysis. Considering the presence of right-censored limits of NT-proBNP levels because of the detection threshold (≤35000 pg/mL), a multiple Tobit linear regression analysis was performed to precisely quantify the association between log-transformed NT-proBNP data and other factors. Potential covariates were entered into the model if they had a univariate P value <0.10. To determine the association between NT-proBNP levels and outcomes, Kaplan–Meier plots by tertiles of NT-proBNP levels were constructed in different body composition strata. Then, Cox proportional hazard analyses were performed to identify the prognostic impact of log NT-proBNP levels stratified according to BMI and body composition. The following variates were included in the multivariate model: age, sex, LVEF, eGFR, Killip >1, TIMI risk score, medical history (previous MI, heart failure, and hypertension) and medications at discharge. The assumption of proportional hazards was assessed on the basis of the methods described by Grambsch and Therneau [15]. Model calibration was evaluated using the Gronnesby and Borgan test [16]. To assess differences between the Cox regression coefficients of NT-proBNP of the body composition strata, the z test was performed as described previously [17]. All statistical analyses were performed using Stata/MP 13.0. A two-side P value <0.05 was considered statistically significant.

## Results

Of 2251 patients with ACS enrolled into the West China Hospital CAD database, 1623 patients had complete data on NT-proBNP and BMI for analysis in the present study. The baseline characteristics of the patients are shown in Table 1. Age was significantly lower among patients with a high BMI or LMI than among those with a low BMI or LMI. In contrast, age and the number of risk factors for coronary artery disease were significantly higher among patients with a high BF amount than among those with a low BF amount.

**Table 1:** Baseline characteristics of patients divided by BMI, LMI, and BF tertiles

	BMI strata				LMI strata				BF strata			
	Tertile 1	Tertile 2	Tertile 3	P value	Tertile 1	Tertile 2	Tertile 3	P value	Tertile 1	Tertile 2	Tertile 3	P value
N	546	539	538		541	542	540		541	541	541	
Age, year (IQR)	69 (60–74)	66 (58–72)	65 (56–72)	<0.001	71 (64–85)	66 (59–72)	61 (58–68)	<0.001	62 (55–70)	67 (59–73)	69 (62–74)	<0.001
Female, n (%)	121 (22.2)	123 (22.8)	119 (22.1)	0.96	121 (22.4)	122 (22.5)	120 (22.2)	1.00	121 (22.4)	121 (22.4)	121 (22.4)	1.00
Diagnosis, n (%)				0.112				0.232				0.079
AMI	242 (44.3)	219 (40.6)	205 (38.1)		238 (44.0)	215 (39.7)	213 (39.4)		243 (44.9)	213 (39.4)	210 (38.8)	
UA	304 (55.7)	320 (59.4)	333 (61.9)		303 (56.0)	327 (60.3)	327 (60.6)		298 (55.1)	328 (60.6)	331 (61.2)	
BMI, kg/m^2^ (IQR)	21.6 (20.3–22.4)	24.1 (23.5–24.7)	27.0 (26.0–28.4)	<0.001	21.6 (20.3–22.5)	24.1 (23.4–24.8)	26.9 (26.0–28.4)	<0.001	21.6 (20.3–22.8)	24 (23.1–24.9)	26.8 (25.7–28.4)	<0.001
LMI, kg/m^2^ (IQR)	16.4 (14.7–17.0)	17.8 (17.2–18.2)	19.0 (18.3–19.7)	<0.001	16.4 (14.7–17.0)	17.9 (17.5–18.2)	19.0 (18.6–19.7)	<0.001	16.6 (15.0–17.5)	17.5 (16.4–18.3)	18.7 (17.5–19.6)	<0.001
BF, % (IQR)	24.3 (21.3–26.2)	26.3 (24.8–28.5)	30.0 (28.5–34.7)	<0.001	24.3 (22.0–27.2)	26.7 (24.5–29.5)	30.0 (27.5–34.7)	<0.001	23.0 (21.3–24.1)	26.1 (25.2–27.2)	30.1 (28.6–34.8)	<0.001
Heart rate, per minute (IQR)	73 (65–82)	73 (65–82)	73 (65–82)	0.627	75 (65–84)	72 (65–82)	73 (65–82)	0.320	73 (65–82)	73 (65–82)	73 (65–82)	0.967
Systolic blood pressure, mmHg (IQR)	127 (110–143)	130 (115–145)	132 (120–146)	<0.001	128 (110–144)	130 (115–145)	130 (118–145)	0.081	124.5 (110–140)	130 (116–146)	134 (120–148)	<0.001
Diastolic blood pressure, mmHg (IQR)	74 (66–82)	75 (70–83)	79 (70–88)	<0.001	72 (65–82)	75 (70–82)	80 (70–89)	<0.001	74 (68–84)	75 (68–84)	78 (70–86)	0.017
Previous MI, n (%)	135 (24.8)	141 (26.4)	141 (26.6)	0.764	139 (25.8)	130 (24.1)	148 (27.7)	0.405	147 (27.2)	131 (24.4)	139 (26.0)	0.574
Previous PCI, n (%)	42 (7.7)	38 (7.1)	42 (7.8)	0.873	41 (7.6)	42 (7.8)	39 (7.3)	0.951	36 (6.7)	43 (8.0)	43 (8.0)	0.656
Previous heart failure, n (%)	26 (4.8)	27 (5.1)	39 (7.3)	0.149	31 (5.8)	27 (5.0)	34 (6.3)	0.639	22 (4.1)	24 (4.5)	46 (8.6)	0.002
Medical history												
Previous CVD, n (%)	21 (3.9)	15 (2.8)	16 (3.0)	0.571	24 (4.4)	14 (2.6)	14 (2.6)	0.141	13 (2.4)	23 (4.3)	16 (3.0)	0.207
Hypertension, n (%)	264 (49.4)	286 (54.0)	320 (60.2)	0.002	271 (51.0)	307 (57.4)	292 (55.0)	0.110	232 (43.8)	285 (53.7)	353 (65.9)	<0.001
Type 1 or 2 diabetes mellitus, n (%)	109 (20.3)	134 (25.1)	131 (24.5)	0.125	118 (22.1)	140 (25.9)	116 (21.7)	0.199	103 (19.4)	124 (23.0)	147 (27.4)	0.008
Current smoking, n (%)	80 (15.4)	84 (16.5)	81 (15.8)	0.886	67 (13.1)	85 (16.6)	93 (18.0)	0.084	98 (19.1)	85 (16.6)	62 (12.1)	0.008
Dyslipidemia, n (%)	62 (12.3)	81 (16.8)	105 (21.6)	<0.001	53 (10.6)	83 (17.2)	112 (22.7)	<0.001	70 (14.1)	76 (15.5)	102 (21.0)	0.01
Laboratory values												
Total cholesterol, mmol/L (IQR)	4.0 (3.4–4.7)	4.0 (3.4–4.9)	4.0 (3.4–4.8)	0.509	3.9 (3.3–4.6)	4.0 (3.4–4.9)	4.0 (3.4–5.0)	0.021	4.0 (3.4–4.8)	4.0 (3.4–4.8)	4.0 (3.4–4.8)	0.917
eGFR, ml/min (IQR)	76.4 (60.8–90.5)	78.2 (62.5–91.8)	77.8 (61.7–91.2)	0.627	72.9 (56.9–86.7)	78.3 (63.6–92.3)	80.7 (64.5–94.1)	<0.001	82.8 (68.5–96.0)	77.3 (61.7–90.6)	73.2 (57.3–85.9)	<0.001
LVEF, % (IQR)	61 (51–68)	62 (51–67)	64 (53–69)	0.017	61 (50–68)	62 (52–68)	64 (53–68)	0.179	61 (51–67)	63 (51–68)	64 (54–68)	0.014
LVEDV, ml (IQR)	101 (83–120)	104 (89–127)	109 (93–129)	<0.001	103 (83–123)	103 (90–124)	108 (92–129)	0.002	102 (83–124)	103 (88–124)	108 (93–128)	0.005
Troponin >100 upper limit of normal, n (%)	135 (25.2)	130 (24.7)	115 (22.0)	0.409	139 (26.2)	120 (22.7)	121 (23.0)	0.324	136 (25.7)	129 (34.3)	115 (21.9)	0.341
Clinical severity of ACS												
Killip >1, n (%)	101 (18.5)	104 (19.3	85 (15.8	0.292	101 (18.7)	105 (19.4)	84 (15.6)	0.219	101 (18.7)	96 (17.7)	93 (17.2)	0.814
TIMI score				<0.001				<0.001				<0.001
Low risk, n (%)	41 (7.5)	98 (18.2)	168 (31.2)		24 (4.4)	80 (14.8)	203 (37.6)		79 (14.6)	105 (19.4)	123 (22.7)	
Medium risk, n (%)	189 (34.6)	204 (37.9)	198 (36.8)		161 (29.8)	222 (41.0)	208 (38.5)		229 (42.3)	171 (31.6)	191 (35.3)	
High risk, n (%)	316 (57.9)	237 (44.0)	172 (32.0)		356 (65.8)	240 (44.3)	129 (23.9)		233 (43.1)	265 (49.0)	227 (42.0)	
Angiographic and interventional data												
Three vessel disease, n (%)	174 (33.4)	147 (28.7)	157 (30.5)	0.258	179 (34.8)	157 (30.1)	142 (27.7)	0.043	158 (30.8)	148 (28.6)	172 (33.2)	0.280
Left main disease, n (%)	65 (12.5)	55 (10.7)	59 (11.5)	0.681	68 (13.2)	55 (10.5)	56 (10.9)	0.345	53 (10.3)	59 (11.4)	67 (12.9)	0.422
One or two vessel disease, n (%)	313 (60.1)	337 (65.8)	327 (63.5)	0.157	300 (58.4)	342 (65.5)	335 (65.4)	0.024	330 (64.3)	332 (64.2)	315 (60.8)	0.412
No. of stents implanted (SD)	1.9 (1.1)	1.9 (1.1)	1.9 (1.1)	0.912	1.9 (1.2)	1.9 (1.1)	1.8 (1.1)	0.221	1.8 (1.1)	1.8 (1.1)	1.9 (1.1)	0.162
Medications at discharge, n (%)												
Aspirin	503 (92.6)	505 (95.1)	500 (93.1)	0.203	495 (92.2)	512 (95.3)	501 (93.3)	0.099	502 (93.5)	505 (94.2)	501 (93.1)	0.747
Clopidogrel	497 (91.5)	500 (94.2)	497 (92.6)	0.246	490 (91.3)	506 (94.2)	498 (92.7)	0.170	502 (93.5)	498 (92.9)	494 (91.8)	0.567
Statin	490 (90.2)	487 (91.9)	491 (91.4)	0.613	484 (90.1)	494 (92.2)	489 (91.2)	0.500	491 (91.4)	487 (91.0)	490 (91.1)	0.968
ACEI/ARB	303 (55.9)	302 (57.0)	319 (59.5)	0.469	306 (57.1)	317 (59.3)	301 (56.1)	0.557	287 (53.5)	313 (58.4)	324 (60.5)	0.064
Beta-blocker	334 (61.6)	336 (63.3)	378 (70.5)	0.005	325 (60.6)	348 (64.9)	375 (69.8)	0.007	334 (62.3)	341 (63.6)	373 (69.5)	0.033
CCB	112 (20.7)	137 (25.9)	170 (31.7)	<0.001	117 (21.9)	146 (27.3)	156 (29.1)	0.020	102 (19.1)	137 (25.7)	180 (33.5)	<0.001
Nitrate	228 (42.1)	234 (44.2)	250 (46.6)	0.319	238 (44.4)	235 (43.9)	239 (44.6)	0.975	203 (37.9)	252 (47.2)	257 (47.9)	0.001

*ACEI* angiotensin-converting enzyme inhibitors; *ACS* acute coronary syndrome; *AMI* acute myocardial infarction; *ARB* angiotensin receptor bloker; *BF* body fat; *BMI* body mass index; *CCB* Calcium channel blockers; *eGFR* estimated glomerular filtration rate; *IQR* interquartile; *LMI* lean mass index; *LVEF* left ventricular ejection fraction; *LVEDV* left ventricular end-diastolic volume; *MI* myocardial infarction; *TIMI* thrombolysis in myocardial infarction; *UA* unstable angina

**Fig. 1:**
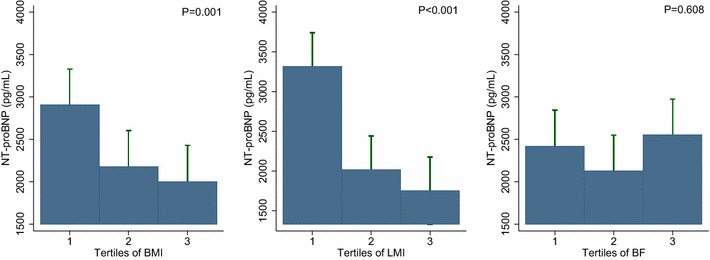
NT-proBNP levels across the BMI (**a**), LMI (**b**), and BF (**c**) groups. The NT-proBNP levels decreased as the BMI (**a**) and LMI (**b**) increased. The NT-proBNP levels did not differ across the BF tertiles (**c**). *NT-proBNP* N-terminal-pro B-type natriuretic peptide; *BMI* body mass index; *LMI* lean mass index; *BF* body fat

The association between NT-proBNP levels and BMI or body composition in the ACS patients is illustrated in Fig. 1. The plasma NT-proBNP levels significantly decreased with the increase in BMI and LMI (P = 0.001 and P < 0.001, respectively); however, no such decrease was noted with the increase in the BF amount (P = 0.608; Fig. 1). In multivariate Tobit linear regression analysis with only BMI as the measure of obesity in model 1, BMI as a continuous variable was inversely associated with log NT-proBNP levels, and when LMI and BF were added into model 1, only LMI showed an independent inverse correlation with NT-proBNP levels. Additionally, similar results were obtained when BMI, LMI, and BF were considered as categorical variables (Table 2).

**Table 2:** Multivariable Tobit linear regression for Log NT-proBNP as dependent variable

	β(SE)	P value
Model 1		
BMI (kg/m^2^)	−0.036 (0.012)	*0.003*
Model 2		
BMI (kg/m^2^)	0.046 (0.081)	0.575
LMI (kg/m^2^)	−0.692 (0.218)	*0.002*
BF (%)	0.154 (0.091)	0.091
Model 3		
BMI		
2nd BMI tertile^a^	−0.240 (0.089)	*0.007*
3rd BMI tertile^a^	−0.259 (0.092)	*0.005*
Model 4		
BMI		
2nd BMI tertile^a^	0.023 (0.155)	0.882
3rd BMI tertile^a^	0.098 (0.236)	0.680
LMI		
2nd LMI tertile^a^	−0.384 (0.147)	*0.009*
3rd LMI tertile^a^	−0.533 (0.213)	*0.012*
BF		
2nd BF tertile^a^	0.024 (0.123)	0.845
3rd BF tertile^a^	0.092 (0.193)	0.633

BMI, LMI, and BF are considered as continuous variables in model 1 and model 2 and considered as categorical variables in model 3 and model 4

Those models are adjusted for age, sex, LVEF, LVEDV, CKD-EPI, Killip >1, heart rate at admission, AMI, peak troponin, and previous MI

Abbreviations as in Table 1

^a^ Reference is the 1st tertile

**Fig. 2:**
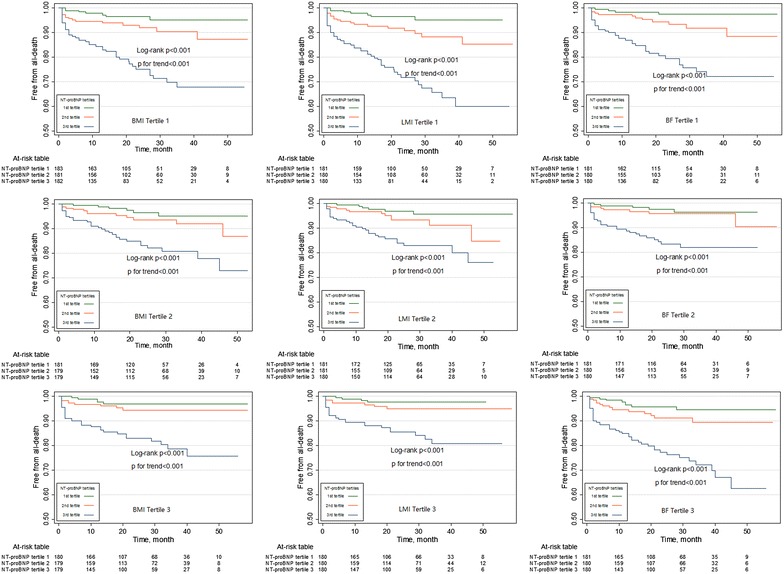
Kaplan–Meier plots according to the NT-proBNP tertiles across the BMI, LMI, and BF tertiles. *Kaplan–Meier* *plots* showing that the NT-proBNP tertiles are associated with an increased risk of mortality in all BMI, LMI, and BF strata. *NT-proBNP* N-terminal-pro B-type natriuretic peptide; *BMI* body mass index; *LMI* lean mass index; *BF* body fat

**Fig. 3:**
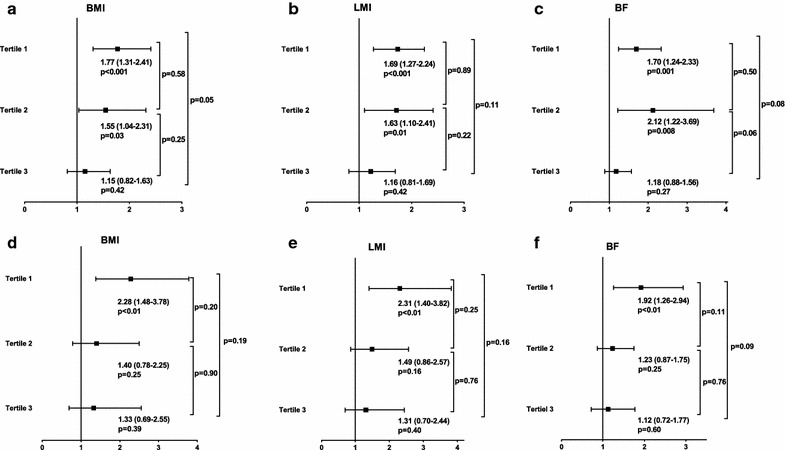
HRs of log NT-proBNP levels considered as continuous variables for both outcomes in the body composition subgroups. The prognostic performance of the NT-proBNP level for all-cause mortality (**a**–**c**) and cardiovascular death (**d**–**f**) is attenuated with increasing BMI, LMI, and BF. *HR* hazard ratio; *NT-proBNP* N-terminal-pro B-type natriuretic peptide; *BMI* body mass index; *LMI* lean mass index; *BF* body fat

Over a median follow-up of 23 months (interquartile range, 16–34 months; maximum follow-up, 5.5 years), 161 all-cause deaths occurred, and of these, 93 (57.8 %) were attributed to cardiovascular causes. Kaplan–Meier plots showed that the NT-proBNP tertiles were associated with an increased risk of mortality in all the BMI, LMI, and BF strata (all log-rank P and P for trend <0.001 in each group; Fig. 2). In terms of all-cause death, multivariate Cox analysis showed that the NT-proBNP level was an independent predictor of mortality in the 1st and 2nd BMI, LMI, and BF tertiles. However, the prognostic value of the NT-proBNP level was not statistically significant in the high BMI, LMI, and BF groups (Fig. 3). The goodness of fit of the multivariate model was satisfactory (P = 0.21). With regard to cardiovascular death, the multivariate Cox analysis showed a significant association between the NT-proBNP level and cardiovascular death among patients with low BMI, LMI, and BF amount, whereas the association was absent among patients with high BMI, LMI, and BF amount (Fig. 3). The goodness of fit of the multivariate model was satisfactory (P = 0.61), and there was no significant violation of the proportional hazards assumption (P = 0.12 for all cause death and P = 0.06 for cardiovascular death).

**Fig. 4:**
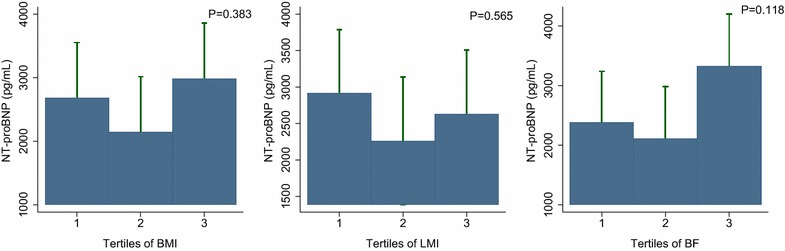
NT-proBNP levels across the BMI (**a**), LMI (**b**), and BF (**c**) groups among the diabetic subgroups. Inverse associations between NT-proBNP levels and increased BMI or body composition are not observed in the diabetic patients. *NT-proBNP* N-terminal-pro B-type natriuretic peptide; *BMI* body mass index; *LMI* lean mass index; *BF* body fat

**Fig. 5:**
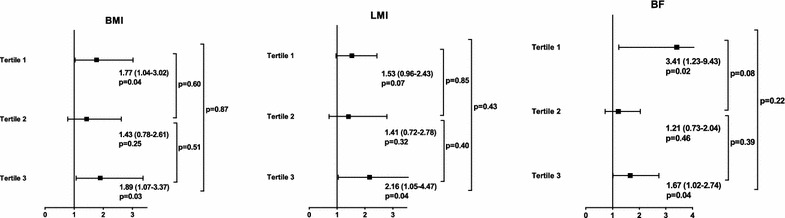
HRs of log NT-proBNP levels considered as continuous variables for all-cause mortality in the body composition subgroup of diabetic patients. The influence of body composition on the prognostic performance of the NT-proBNP level for mortality is attenuated in the diabetic subgroups. *HR* hazard ratio; *NT-proBNP* N-terminal-pro B-type natriuretic peptide; *BMI* body mass index; *LMI* lean mass index; *BF* body fat

In the subgroup of patients with diabetes, inverse associations between NT-proBNP levels and BMI or body composition were not observed (Fig. 4). In addition, the negative influence of high BMI and body composition on the prognostic performance of the NT-proBNP level appeared to be attenuated (Fig. 5).

## Discussion

The present study found an inverse association between BMI and NT-proBNP levels in patients with ACS. Furthermore, when body composition was considered, LMI, but not BF, was inversely associated with NT-proBNP levels. The prognostic performance of the NT-proBNP level in patients with ACS was compromised among patients with high BMI, LMI, or BF. However, the influence of BMI and body composition on the NT-proBNP level and its prognostic performance appeared to be attenuated in diabetic patients with ACS. To our knowledge, this is the first study to investigate the association between body composition and the prognostic performance of the NT-proBNP level in patients with ACS.

Myocardial ischemia and hypoxia have been shown to directly induce an increase in the expression of proBNP, even in the absence of left ventricular dysfunction [18]. It has been reported that NT-proBNP levels are high across the entire spectrum of CADs and the increase in associated with CAD severity [19, 20]. Additionally, the angiographic severity of CAD has been shown to be better reflected by the NT-proBNP level than the BNP level [21]. Several studies have validated the prognostic performance of the NT-proBNP level in patients with ACS, which was independent of LVEF, troponin, and renal insufficiency [2, 3]. Thus, NT-proBNP has been proposed as a promising biomarker for the early diagnosis of ACS [22] and its risk stratification [23–25].

However, the true implications of NT-proBNP in clinical practice were typically influenced by confounders, such as age, sex, diabetes, and renal function. Recently, a study showed that BMI was inversely associated with BNP/NT-proBNP levels [4]. Some of the possible explanations for this phenomenon include an increase in the clearance of natriuretic peptide by the C-type natriuretic peptide receptor [26], impairment of the natriuretic peptide response termed “natriuretic peptide handicap” [27], and compromised detection of NT-proBNP in obese individuals [28]. However, some of these hypotheses have been contradicted [11]. Therefore, the exact mechanism underlying the negative association between NPs and obesity remains undefined. A previous study proposed a bidirectional relationship between BNP and adiposity because BNP has been shown to cause lipolysis via A-type natriuretic peptide receptor [5]. Additionally, Li et al. [29] recently suggested that changes in liraglutide-induced lean mass and body fat were associated with increases in plasma NP levels in obese type 2 diabetic patients. However, our finding that the NT-proBNP levels differ across LMI tertiles, but not BF tertiles, in patients with ACS does not support this hypothesis. In fact, previous studies by Das et al. and Oreopoulos et al. [8, 11] showed that a high LMI, but not BF, was associated with low BNP/NT-proBNP levels in both the general population and a chronic heart failure population. In the present study, these findings were extended to patients with ischemic heart disease. Chang et al. provided a plausible explanation for this finding. The authors demonstrated that free testosterone was a mediator of the association between lean mass and NPs through an increase in lean mass and a decrease in NP synthesis; however, estradiol, which plays an important role in the regulation of adiposity [30], does not appear to influence NP levels [31].

Although numerous studies focused on the association between BMI and NT-proBNP levels, few studies have evaluated whether BMI influences the prognostic performance of NT-proBNP in heart diseases, especially ACS. Choi et al. performed a study of 2736 Koreans with acute myocardial infarction (AMI) and showed that NT-proBNP levels were lower in obese AMI patients than in non-obese AMI patients and that NT-proBNP was an independent prognostic factor in obese AMI patients. However, it should be pointed out that these authors did not assess the goodness of fit and overall performance of the logistic regression models [7]. Considering that NT-proBNP in the multivariate model unreasonably failed to predict outcomes in the normal group (P = 0.113), the calibration of the model was very likely to not be good. Thus, the results were not convincing. On the contrary, Lorgis et al. addressed this issue in a prospective study of 2217 AMI patients. The authors used the WHO BMI classification system to classify the extent of obesity in the patients and showed that log NT-proBNP independently predicted 1-year cardiovascular mortality in normal and overweight patients. However, in obese patients, the propeptide levels failed to retain their prognostic value (OR = 1.34, P = 0.244). The goodness of fit of the final model was good [−2log-likelihood: 642; P (Hosmer–Lemeshow) = 0.479] [6]. Our results from the BMI subgroups extended the findings of the study by Lorgis et al. to the entire spectrum of ACS covering unstable angina.

It is believed that low BNP/NT-proBNP levels are good for prognosis [10]. As obese patients have low BNP/NT-proBNP levels, this perspective appears to mirror the obesity paradox in which obesity has a paradoxical survival benefit [32]. However, the paradox has been almost exclusively documented in studies that use BMI as a measure of obesity. Oreopoulos et al. [8] suggested that BMI, as a surrogate for obesity, was better correlated with lean mass than body fat. Obesity indexed by BMI may sometimes indicate excessive muscle rather than excessive fat. Additionally, a previous study has suggested that lean mass, but not body fat, can predict all-cause death in patients with coronary artery disease [12]. Therefore, in the present study, we divided patients according to body compositions in order to accurately evaluate the influence of true obesity on the prognostic performance of NT-proBNP. Low NT-proBNP levels were observed in patients with high LMI, but not in those with high BF, and the prognostic performance of NT-proBNP was significantly compromised in patients with high LMI and high BF. These findings suggest that it is not plausible to simply assume that low NT-proBNP levels reflect mild disease severity in obesity. In fact, early studies have suggested that BNP/NT-proBNP levels failed to reflect LV filling pressure in obese individuals, indicating that BNP/NT-proBNP should not be considered as a surrogate for disease severity in obese patients [33].

Obesity is typically accompanied with diabetes. There is growing evidence suggesting that low NP levels might play a role in the development of future diabetes [34]. In contrast, established diabetes has been shown to increase BNP/NT-proBNP levels [35], which is partially attributed to diabetes-associated cardiac remodeling. Recent studies have shown that diabetes is an important extra-cardiac parameter that is associated with changes in BNP/NT-proBNP levels and that a high NT-proBNP level is a strong predictor of mortality in patients with diabetes [35, 36]. Therefore, diabetes and body composition exert opposite effects on BNP and NT-proBNP changes, and the interpretation of NT-proBNP levels in diabetic and obese individuals is complicated. It is interesting to assess the influence of body composition on the NT-proBNP level and its prognostic performance in diabetic patients. Lunchner et al. [37] demonstrated that baseline established diabetes, but not BMI, was independently associated with changes in BNP/NT-proBNP levels during a 10-year follow-up. From a dynamic perspective, this finding indicates that the effect of longstanding diabetes on the BNP/NT-proBNP level will gradually become greater than the effect of body composition on the BNP/NT-proBNP level, which is a plausible explanation for our finding that diabetes attenuated the influence of body composition on the NT-proBNP level and its prognostic value. Unfortunately, we were unable to accurately evaluate the effect of diabetes duration on NT-proBNP changes in obese individuals, as data on the diabetes duration were unavailable. Future studies are needed to address this issue.

The present study had several limitations. First, although this study focuses on the relationship between body composition and NT-proBNP levels, body fat and lean mass were not directly measured. Thus, further studies in which lean mass and body fat are directly measured using methods, such as dual-energy X-ray absorptiometry and bioelectrical impedance, are urgently needed. Nevertheless, the CUN-BAE equation has been derived from a large sample and validated in another large population. Meanwhile, as direct measurement of BF and lean mass is rarely applied in clinical practice, assessment of body composition using equations, such as the CUN-BAE equation, probably reflects the situation in a routine clinical setting. Second, despite the large sample size, the present study was a single-center observational study. Therefore, confounding factors could not be entirely avoided. Third, potential selection bias might have been present, as this is an inherent limitation of real-world studies. However, considering the relatively large sample size, the results were not likely to be by chance. Forth, the number of diabetic patients was small, and this might have cause the insufficient results. Further studies in a larger population are needed to overcome this issue. Fifth, the study population mainly included Chinese individuals, and thus, we cannot extrapolate the findings to other races/ethnicities. A previous study reported that the NT-proBNP levels vary across different races [38].

## Conclusion

In a large population of >1600 patients with ACS, we confirmed that BMI and LMI, but not BF amount, are inversely associated with NT-proBNP levels. Additionally, the prognostic performance of this biomarker may be compromised in patients with high BMI, LMI, or BF. The influence of body composition on the NT-proBNP level and its prognostic performance might be attenuated in diabetic patients with ACS.

## Abbreviations

ACS: acute coronary syndrome
AMI: acute myocardial infarction
BF: body fat
BMI: body mass index
CAD: coronary artery disease
CKD-EPI: chronic kidney disease epidemiology
HR: hazard ratio
LMI: lean mass index
LVEF: left ventricular ejection fraction
LVEDV: left ventricular end-diastolic volume
NP: natriuretic peptide
NT-proBNP: N-terminal-pro B-type natriuretic peptide
TIMI: thrombolysis in myocardial infarction
